# *Coxiella burnetii* as a possible cause of autoimmune liver disease: a case report

**DOI:** 10.4076/1752-1947-3-8870

**Published:** 2009-08-10

**Authors:** Chloe Kaech, Isabelle Pache, Didier Raoult, Gilbert Greub

**Affiliations:** 1 Division of Infectious Diseases, Centre Hospitalier Universitaire Vaudois, 1011 Lausanne, Switzerland; 2 Division of Gastroenterology, Centre Hospitalier Universitaire Vaudois, 1011 Lausanne, Switzerland; 3 Unité des Rickettsies, Faculté de Médecine, Université de la Méditerranée, Marseille, France; 4 Institute of Microbiology, University of Lausanne, Lausanne, Switzerland

## Abstract

**Introduction:**

Q fever is a zoonotic infection that may cause severe hepatitis. Q-fever hepatitis has not yet been associated with autoimmune hepatitis and/or primary biliary cirrhosis.

**Case presentation:**

We describe a 39-year-old man of Sri Lankan origin with chronic Q-fever hepatitis who developed autoantibodies compatible with autoimmune hepatitis/primary biliary cirrhosis overlap syndrome. Ursodeoxycholic acid in addition to antibiotic therapy markedly improved hepatic enzyme levels suggesting that autoimmunity, potentially triggered by the underlying infection, was involved in the pathogenesis of liver damage.

**Conclusion:**

We suggest that *Coxiella burnetii* might trigger autoimmune liver disease. Patients with Q-fever hepatitis who respond poorly to antibiotics should be investigated for serological evidence of autoimmune hepatitis, primary biliary cirrhosis or overlap syndrome, as these patients could benefit from adjunctive therapy with ursodeoxycholic acid. Conversely, *C. burnetii* serology might be necessary in patients with autoimmune liver disease in order to exclude underlying *Coxiella* infection.

## Introduction

Q fever is a zoonotic infection that may cause severe hepatitis. Q-fever hepatitis has not yet been associated with autoimmune hepatitis and/or primary biliary cirrhosis.

## Case presentation

We report the case of a patient with chronic Q-fever hepatitis with autoantibodies suggestive of autoimmune hepatitis/primary biliary cirrhosis (AIH/PBC) overlap syndrome. A previously healthy 39-year-old man of Sri Lankan origin presented at the emergency department of the university hospital of Lausanne, Switzerland in March 2005, complaining of fever, dry cough, nausea, vomiting, and headache. The patient, who had lived in an urban area of Switzerland without animal contact for the previous 17 years, had returned from a one month stay in rural Sri Lanka 14 days before the onset of symptoms. On physical examination, he was febrile at 39°C with stable vital signs and no apparent site of infection. Laboratory analyses showed normal leukocyte, hemoglobin and platelet levels, but elevated values for creatinine (117 μmol/L), C-reactive protein (75 mg/L), alanine aminotransferase (ALAT, 120 U/L), gamma-glutamyl transferase (γ-GT, 240 U/L) and bilirubin (27 μmol/L). A test for malaria was negative. Lumbar puncture, chest X-ray and abdominal ultrasound results were unremarkable. Treatment with doxycycline was administered for 10 days for presumptive leptospirosis with rapid resolution of clinical signs and symptoms, but with persistently elevated liver enzymes. Blood cultures remained sterile, and serology was negative for leptospirosis, rickettsiosis, HIV and hepatitis A, B and C. Q-fever serology was not carried out.

One month later, the patient's symptoms recurred. Serological testing was compatible with Q-fever in transition from acute to chronic disease, with phase I antibodies and high titers of phase II antibodies being present (phase I: immunoglobulin (Ig)G, 1600; IgA, 80; IgM, 320; phase II: IgG, 25,600; IgA, 640; IgM, 640). Because of the persistent hepatic cytolysis and cholestasis (ALAT 157U/L, γ-GT 546U/L), a liver biopsy was carried out which revealed a mixed inflammatory infiltrate associated with small non-necrotising epitheloidal cell granulomas without giant cell formation, lipid vacuoles or fibrinoid rings. A diagnosis of Q-fever hepatitis was made and the patient was referred to our infectious diseases service in July 2005.

**Figure 1:**
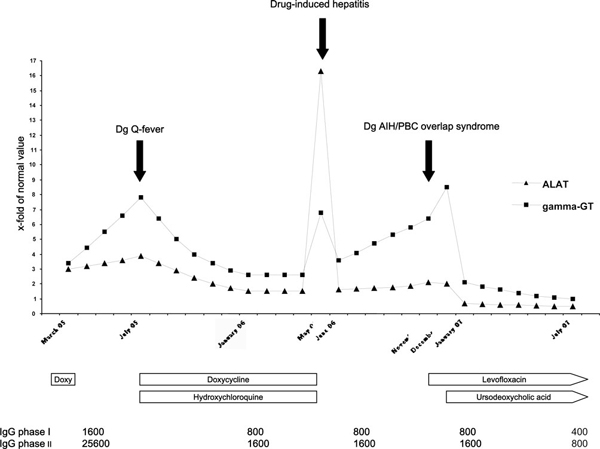
**Changes in hepatic enzyme levels and Q-fever serology over time and their correlation with therapy**. Numbers on the y-axis are x-fold of normal value (40 U/L for alanine aminotransferase; 70 U/L for gamma-glutamyl transferase). IgG G phase I/II: IgG G antibodies against *C. burnetii* phase I and II antigen.

At this stage, the patient was afebrile and no longer had cough, vomiting or headache, but complained of persistent fatigue and intermittent right upper quadrant abdominal pain. Hematological parameters, serum creatinine, C-reactive protein and bilirubin were normal. Echocardiography showed normal systolic function without valvular regurgitation or vegetations. Therapy with doxycycline 200 mg/day and hydroxychloroquine 600 mg/day was initiated, with improvement in the patient's symptoms, hepatic enzyme values (ALAT 60 U/L, γ-GT 179 U/L) and antibody titers against *Coxiella burnetii* (phase I: IgG, 800; IgA, 0; IgM, 0; phase II: IgG, 1600; IgA, 0; IgM, 0) within 6 months of therapy (Figure 1).

In May 2006, an acute increase in hepatic enzyme levels was attributed to toxic hepatitis induced by either doxycycline or hydroxychloroquine (ALAT 653 U/L, γ-GT 467 U/L). A second liver biopsy, showing a mixed inflammatory infiltrate with non-confluent hepatocellular necrosis and without granulomas, supported the hypothesis of drug toxicity. Interruption of doxycycline and hydroxychloroquine therapy resulted in a rapid improvement of hepatic cytolysis and cholestasis (ALAT 63 U/L, γ-GT 252 U/L). Treatment was temporarily withheld because of concerns about repeated drug-induced liver injury.

During the following months, the patient's liver enzymes remained stable, so we reintroduced antibiotic therapy with levofloxacin 1g/day in November 2006. Given a progressive discrepancy in ALAT and γ-GT levels (ALAT 85 U/L, γ-GT 451 U/L), the possibility of an additional liver disease was considered. Abdominal ultrasound showed a discrete hepatomegaly without dilatation of the biliary tract. First-time screening for autoimmune liver disease revealed smooth muscle antibodies (SMA)+++, an antinuclear antibody (ANA) titer of 1:320, antimitochondrial antibodies (AMA)++, an anti-M2 antibody titer of 1:1140, and a total serum IgM level of 8.5 g/L (0.3-2.4 g/L), consistent with autoimmune hepatitis/primary biliary cirrhosis (AIH/PBC) overlap syndrome, which is a rare condition in which patients have features of both diseases. Hepatic cytolysis and/or cholestasis are present. Histological findings may be non-specific or typical of either AIH or PBC. Serology shows markers of AIH (ANA, SMA) and of PBC (AMA, anti-M2 antibodies, elevation of total IgM levels) [1,2]. Treatment consists of ursodeoxycholic acid alone or in combination with corticosteroids [3]. In our patient, a therapeutic trial with ursodeoxycholic acid was initiated. While liver function tests had not improved after 1 month of levofloxacin (ALAT 80 U/L, γ-GT 596 U/L), a dramatic decrease in both ALAT and γ-GT levels was observed after 1 month of ursodeoxycholic acid (ALAT 27 U/L, γ-GT 147 U/L) therapy. By July 2007, with ongoing levofloxacin and ursodeoxycholic acid treatment, the patient had less fatigue and intermittent abdominal pain. His liver enzymes were normal (ALAT 18 U/L, γ-GT 67 U/L), and antibody titers against *C. burnetii* had decreased (phase I: IgG, 400; IgA, 0; IgM, 0; phase II: IgG, 800; IgA, 0; IgM, 0). Levofloxacin therapy will be continued until the phase I IgG titer is below 400, with a minimum treatment duration of 18 months.

## Discussion

We report the simultaneous presence of chronic Q-fever hepatitis and autoimmune liver disease. Serology for *C. burnetii* is highly specific, and we are not aware of any reports of false-positive Q-fever serology in the context of autoimmune disease. Moreover, liver enzymes and *C. burnetii* serology clearly improved with antibiotic therapy, leaving no doubt that our patient really had Q-fever hepatitis.

Autoantibodies are commonly found in both acute and chronic Q-fever [4,5]. Rheumatoid factor, ANA, AMA, SMA, antiphospholipid antibodies, and a positive Coombs test have been reported. It is not clear whether these autoantibodies are an epiphenomenon without clinical significance or if they are instrumental in the pathogenesis of Q-fever. Levy *et al*. [6] described several patients with acute Q-fever and positive SMA who remained febrile despite adequate antibiotic treatment and became afebrile with corticosteroid treatment, suggesting a direct role of autoimmunity in causing the patients' illness. Our patient had autoantibodies compatible with AIH/PBC overlap syndrome and his clinical and laboratory condition strikingly improved following the introduction of ursodeoxycholic acid, which improves liver function in PBC without being beneficial for cholestasis in general [7].

Our patient could simply have developed two rare unrelated liver pathologies at once. Since he was completely asymptomatic before infection with *C. burnetii*, we do not believe that he had pre-existing AIH/PBC overlap syndrome. However, one disease may have triggered the other. AIH/PBC overlap syndrome as a trigger of Q-fever hepatitis is not biologically plausible. On the other hand, *C. burnetii* as a trigger of liver-directed autoimmunity seems plausible. Many autoimmune diseases, such as acute rheumatic fever and Guillain-Barré syndrome, are believed to be induced by an infection through molecular mimicry or immunomodulation [8]. Although AIH clearly shows a genetic, human leukocyte antigen-linked predisposition, there has been evidence implicating hepatitis viruses, measles virus, cytomegalovirus and Epstein-Barr virus as disease triggers [9]. Several features suggest a causal relationship between PBC and infection, such as case clustering within well-demarcated geographical areas and an increased risk of recurrence after liver transplantation with increasing immunosuppression [10]. *Escherichia coli*, atypical mycobacteria and retroviruses have been implicated as causative agents. Roesler *et al*. [11] identified a non-species-specific bacterial protein (β-subunit of bacterial RNA polymerase) as an antibody target in AIH and PBC, suggesting that not one, but many bacterial species might potentially trigger liver-directed autoimmunity.

To our knowledge, there are no reports in the literature that suggest an association between Q-fever hepatitis and autoimmune liver disease. We hypothesize that our patient had Q-fever hepatitis at first, and when antibiotic therapy was interrupted because of drug-induced hepatitis, the sustained presence of *C. burnetii* in the liver triggered a clinically relevant AIH/PBC overlap syndrome by mechanisms yet to be elucidated. Introduction of ursodeoxycholic acid improved hepatic enzyme levels by acting on the autoimmune component of liver disease, whereas antibiotic therapy adequately treated the infectious component, as demonstrated by decreasing antibodies against *C. burnetii*.

## Conclusion

*C. burnetii* might trigger autoimmune liver disease. Patients with Q-fever hepatitis who respond poorly to antibiotics should be investigated for serological evidence of AIH, PBC or overlap syndrome, as these patients could benefit from adjunctive therapy with ursodeoxycholic acid. Conversely, *C. burnetii* serology might be worth doing in patients with autoimmune liver disease in order to exclude underlying *Coxiella* infection.

## Abbreviations

AIH: autoimmune hepatitis; ALAT: alanine aminotransferase; AMA: antimitochondrial antibodies; ANA: antinuclear antibodies; PBC: primary biliary cirrhosis; SMA: smooth muscle antibodies; γ-GT: gamma-glutamyl transferase.

## Consent

Written informed consent was obtained from the patient for publication of this case report and any accompanying images. A copy of the written consent is available for review by the Editor-in-Chief of this journal.

## Competing interests

The authors declare that they have no competing interests.

## Authors' contributions

CK wrote the first draft of the manuscript. All authors contributed to patient care and all corrected and improved the manuscript, and read and approved the final manuscript.
